# Hierarchical Network-Augmented Hydroglasses for Broadband Light Management

**DOI:** 10.34133/2021/4515164

**Published:** 2021-01-20

**Authors:** Zhouyue Lei, Baohu Wu, Peiyi Wu

**Affiliations:** ^1^State Key Laboratory for Modification of Chemical Fibers and Polymer Materials, College of Chemistry, Chemical Engineering and Biotechnology, Center for Advanced Low-Dimension Materials, Donghua University, Shanghai 201620, China; ^2^John A. Paulson School of Engineering and Applied Sciences, Harvard University, Cambridge, MA 02138, USA; ^3^Jülich Centre for Neutron Science (JCNS) at Heinz Maier-Leibnitz Zentrum (MLZ) Forschungszentrum Jülich, Lichtenbergstr. 1, 85748 Garching, Germany

## Abstract

Light management is essential for military stealth, optical information communication, and energy-efficient buildings. However, current light management materials face challenges of limited optical modulation range and poor mechanical properties. Herein, we report a locally confined polymerization (LCP) approach to develop hierarchical network-augmented hydroglasses (HNAH) based on poly(methacrylic acid) for broadband light management as well as mechanical enhancement. The dynamic geometry of the networks ranging from nano- to micro-scale enables to manage the light wavelength over three orders of magnitude, from the ultraviolet (UV) to infrared (IR) band, and reversibly switches transmittance in the visible region. A smart hydroglass window is developed with elasticity, outstanding robustness, self-healing, notch resistance, biosafety by blocking UV radiation, and high solar energy shielding efficacy with a temperature drop of 13°C. Compared to current inorganic glasses and Plexiglas, the hydroglass not only is a promising and versatile candidate but also provides novel insights into the molecular and structural design of broadband light management and optimized mechanical properties.

## 1. Introduction

Broadband light management plays a critical role in military stealth, optical information communication, and energy-efficient buildings [1–4]. However, traditional optical glasses, from inorganic glasses to Plexiglas, are brittle, scratch vulnerable, and rarely auto-regulate the optical properties to adapt to the environmental changes. Recently developed light responsive materials, including metal oxides and organic thermochromic/electrochromic composites, can modulate light only in a narrow band and are limited by the mechanical fragility and complex processing requirements [5]. The optical and mechanical bottlenecks are mainly due to the monotonous light-responsive sites and single-atom bridged networks. For example, transition metal oxides switch between transparent and opaque states during faradic charge injection/extraction at the atomic level [6], liquid crystals change the visible transmittance by modulating the mesoscopic order degree [7, 8]; stimuli-responsive hydrogels show reversible phase separation in the range of hundreds of nanometers [9–11]. None of them achieves a synergy of hierarchical structures. Besides, the energy required for breaking a layer of atomic bonds is only about 1 J m^−2^ and this is the major reason for the brittleness of traditional optical glasses [12]. In fact, for the next generation of versatile glasses toward a broad application prospect including biosafety, military stealth, and energy saving, it is vital to extend the light management ability down to ultraviolet (UV) and up to infrared (IR) regions and also optimize the mechanical properties with elasticity and toughness. Unfortunately, up to now, rare single-component materials have reached such a wide modulation spectrum ranging from nano- to micro-scale. People have to integrate multiple materials and compromise to sophisticated multilayer processing [13].

Herein, we develop a hierarchical network-augmented hydroglass (HNAH) by modifying the chemical structure of Plexiglas for achieving amphiphilicity. The ester group in Plexiglas is substituted by the carboxyl group while the *α*-methyl is retained. Due to the amphiphilicity, liquid-liquid separation emerges in the precursor monomer solution, and thus, we can engineer hierarchical networks in the hydroglass via locally confined polymerization (LCP). The LCP is distinct from conventional polymerization that the single chain polymerizes and crosslinks in homogenous precursors. It enables to polymerize hierarchically phase-separated networks in locally confined amphiphilic domains and thus to optimize the optical and mechanical properties. The resulting HNAH is physically crosslinked by the polymer domains ranging from nano- to micro-scale. It can reversibly switch the transmittance in the visible solar region, block light in the UV and IR spectra, and self-heal to resist scratches and notches. Moreover, molecular mechanisms and structural evolution behind the LCP and dynamic response of the HNAH are also analyzed from the perspective of multidimensional spectroscopy.

## 2. Results

### 2.1. The Origin of the Hierarchical Structures

**Figure 1:**
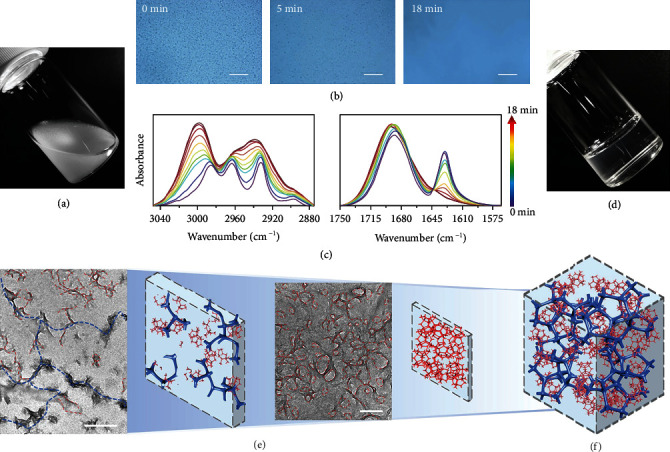
The hierarchical structures of the hydroglass. (a) The photograph of the monomer aqueous solution (MAA doped with about 550 mM positive charge concentration of the trivalent aluminium cation). (b) Optical micrographs of the monomer aqueous solution during the LCP process. Scale bar: 100 *μ*m. (c) The time-dependent FTIR results of the monomer aqueous solution during the LCP process. D_2_O was used as the solvent to eliminate the influence of *δ*(O-H) band of water at about 1640 cm^−1^. (d) The photograph of the HNAH. (e) The cryosection micrographs of the HNAH with different slice thickness for the observation of micronetworks and nanonetworks (from left to right) and their schematic models. Bule and red dash lines indicate the micro- and nanonetworks in the HNAH, respectively. The scale bars are 0.5 *μ*m and 200 nm, respectively. (f) Schematic models of the hierarchical networks of the HNAH.

With the substituted carboxyl group, the hydroglass monomer, i.e., methacrylic acid (MAA), is consisted of a hydrophobic *α*-methyl group and a hydrophilic carboxylic acid group. The amphiphilicity enables to fabricate a multiphase aqueous system with desired structures [14]. The MAA aqueous solution is homogenous and transparent because dominant hydrogen bonds between the monomers and water molecules promote the dispersion and dissolution of monomer (Figure S1). With the addition of counterions (cations with the opposite charges of –COO^−^), hydrogen bonds between carboxylic acid and water molecules tend to partially disassociate, and the generation of associated ionic carboxylate enhances the hydrophobicity. When the charge concentration of the counterions is higher than a critical value, liquid-liquid phase separation emerges in the aqueous monomer solution. For example, Figure 1(a) shows a photograph of the MAA monomer solution with about 550 mM positive charge concentration of a trivalent aluminium cation. The hydrated MAA droplets are uniformly dispersed with diameters of several microns. In this binary system, hydrophobic *α*-methyl and associated ionic carboxylate induce local enrichment of the monomers inside the droplets at the nanoscale, while hydrophilic carboxylic acid guarantees the stable microscale boundaries of the droplets. LCP simultaneously occurs inside and at the boundary of the droplets. During the LCP process, the apparent liquid-liquid phase separation interface gradually disappears (Figure 1(b)) [15]. From the perspective of real-time attenuated total reflectance (ATR) spectra, this is probably because the *α*-methyl/methylene are more hydrated in polymer chains and narrow the difference in refractive index between polymers and water (Figure 1(c), detailed analysis is available in Note S1). The final hydroglass is highly transparent in the visible region at room temperature (Figure 1(d)). The transmittance at 550 nm achieves about 95% (Figure S2), which is higher than conventional inorganic glasses (80-93%) and Plexiglas (92%) [16].

The hydroglass is sectioned into slices of different thicknesses for the observation of microscale and nanoscale structures (transmission electron microscopy (TEM) imaging, Figure 1(e)). The thick slice with low magnification shows the micronetworks with the diameters of about several microns, while the thinner cryosection with high magnification reveals nanonetworks of about 100 nm diameter. The field emission scanning electron microscope (FESEM) image also confirms the hierarchical networks (Figure S3). Micronetworks at a few to a dozen micron scale are observed, and many thin-walled nanoscale networks are located inside. Therefore, as illustrated schematically in Figure 1(f), although the HNAH is transparent in the visible band at room temperature, the hierarchical networks ranging from nano- to micro-scale have impact on the wavelength below a few hundred nanometers and above a few microns. It is different from the conventional transparent materials synthesized from homogeneous precursors where polymer chains grow evenly at the nanoscale.

It is worthwhile to note that, before taking the TEM and FESEM photographs, the aqueous material has to undergo a freezing process. Even though we try to make the hydroglass frozen flash, the growing ice crystals inevitably squeeze the networks and may impact their delicate structures. For in situ observation of the hierarchical networks, small-angle X-ray scattering (SAXS) and very small-angle neutron scattering (VSANS) experiments are also performed, and the results are discussed in the section of structural evolution analysis.

### 2.2. Stretchability, Self-Healing, and Notch-Insensitivity

**Figure 2:**
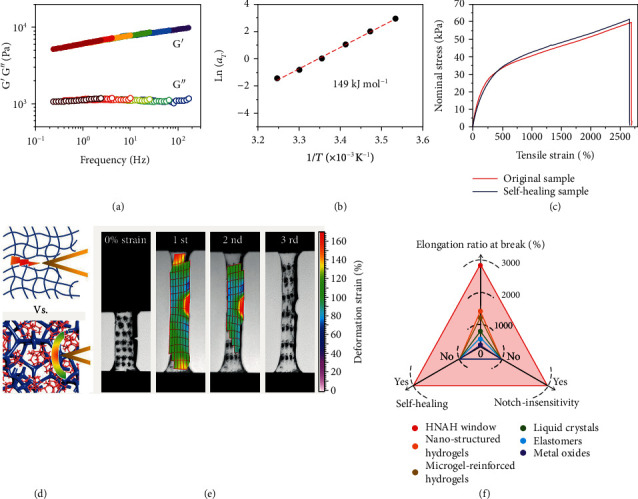
Mechanical properties of the hydroglass. (a) Frequency dependent storage modulus (G′) and loss modulus (G^″^) moduli of the HNAH. (b) Time–temperature horizontal shift factor (*a*_*T*_) derived from the Arrhenius equation. (c) Nominal stress-strain curves of the original HNAH and the self-healing sample. (d) Schematic illustration of the notch-sensitive behavior of conventional glasses crosslinked by single-layer of chemical bonds and notch-insensitive behavior of the HNAH crosslinked by local polymer aggregation domains. (e) Cyclic stretching a notched HNAH sample and the strain mapping. (f) A comparison among this work and previously reported smart windows based on nanostructured hydrogels [28], microgel-reinforced hydrogels [28], liquid crystals [7], elastomers [27], and metal oxides [6].

Although there is no chemical crosslinking agent, the hydroglass is physically crosslinked by hydrogen bonds among carboxylic acid groups, ionic associations between the carboxylate groups and the inorganic counterions, and hydrophobic interactions introduced by *α*-methyl groups [14, 17]. The hierarchical network-augmented structures have high energy barriers to trap the polymer chains and thus reinforce the mechanical properties. The HNAH shows solid-like behavior (G′ > G^″^) in the frequency range of 0.1-200 Hz and the temperature range of 10-35°C (Figure 2(a)). On the basis of time-temperature superposition, the apparent activation energy (*E*_a_) is calculated to be 149 kJ mol^−1^ from Arrhenius equation (Figure 2(b) and detailed calculation are available in Note S2) [18, 19]. It is higher than that of conventional supramolecular networks (41-96 kJ mol^−1^) [19]. The HNAH shows high Young's modulus of about 20.5 kPa and high stretchability up to 2690% elongation ratio at the break (the red line in Figure 2(c)), which is much better than chemically crosslinked networks [20–22]. Owing to the dynamic feature of physically crosslinked networks, a fractured HNAH autonomously self-heals within 48 h and almost recovers the mechanical strength and stretchability (the blue line in Figure 2(c)). Moreover, compared to conventional glasses crosslinked by single layer of chemical bonds, which usually cause concentrated stresses in a notched region, the HANH crosslinked by locally polymer-rich domains requires more energy for fracture and helps to resist crack growth in a notched sample (Figure 2(d)) [23–25]. This is confirmed by digital image correlation (DIC) analysis on a notched HNAH under cyclic loads. The crack is indeed blunted, and the concentrated stress/strain at the crack tip is dissipated to resist the crack propagation (Figure 2(e)) [26].

These results suggest that the HNAH is tough and notch-insensitive. Such impressive robustness and self-healing ability are superior to current optical materials or smart windows. Most of the light management materials, such as the inorganic glasses doped with transition metal oxides, are brittle, easy to broken, and cannot repair the scratches [6]. Even for organic materials, including thermochromic elastomers, electrochromic liquid crystals, and nanostructured/microgel-reinforced hydrogels, they are also limited in stretchability and rarely self-healing [7, 10, 27, 28]. In contrast, the HNAH window with the hierarchical networks ranging from nano- to micro-scale exhibits the advantages of the mechanical performance, which combines stretchability, self-healing, and notch-insensitivity.

### 2.3. Dynamic Light Regulation in the Visible Region

**Figure 3:**
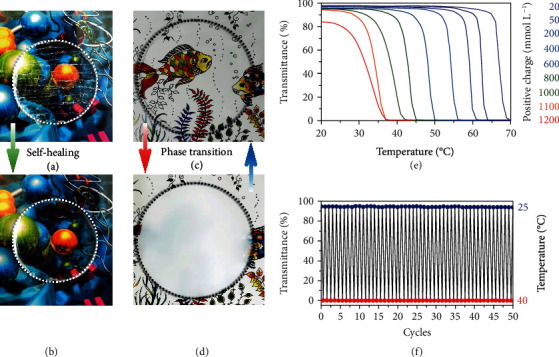
Dynamic light regulation of the hydroglass. (a) A photograph of the HNAH with scratches and notches (outlined with a dotted line). (b) A photograph of the self-healing HNAH (outlined with a dotted line). (c) A photograph of the HNAH window at room temperature (outlined with a dotted line). (d) A photograph of the HNAH window after phase transition (outlined with a dotted line). (e) Transmittance vs. temperature for the HNAH with different cation concentrations (AlCl_3_). (f) Cyclic heating-cooling tests of the HNAH.

As shown in Figure 3(a) and (b), the HNAH window could not only recover its mechanical properties from the fractured sample but also self-repair its optical performance after being scratched and cut off. The transmittance autonomously recovers within 48 h. Moreover, owing to the amphiphilicity of the PMAA, the HNAH window can tune the transmittance in the visible band. The phase transition temperature of an HNAH window with about 550 mM positive charge concentration of the trivalent aluminium cation is 34°C determined by the turbidity test (Figure 3(c) and (d)). It remains almost unchanged when the heating rate changes from 0.1 to 2°C min^−1^, indicating the phase transition behaviour is quite reliable in the suitable time scale (Figure S4). With the decreasing ionic strength, the hydrophilicity of the system is enhanced, and the phase transition temperature shifts to a higher temperature (Figure 3(e)). When the aluminium cation concentration decreases to 20 mM, the phase transition temperature rises to 67°C.

On the other hand, with the increasing ionic strength, the hydrophobicity is enhanced, and the phase transition temperature shifts to a lower temperature. The phase transition temperature is down to 32°C when the aluminium cation concentration increases to 600 mM. Further increasing the cation concentration, the hydroglass becomes opaque at room temperature. It is probably because the local aggregation of polymer chains increases on the nanoscale and the scattering diameters may be up to several hundreds of nanometers. The visible light is thus totally blocked. For the HNAH window with the phase transition temperature of about 34°C, it is suitable for energy-saving buildings. In order to prevent dehydration and increase the lifetime of the HNAH, suitable airtight encapsulation or superhydrophobic self-healing coatings is recommended for practical use [29–31]. For example, when the HNAH is sandwiched between two slides of PE and sealed by epoxy, the weight (water content) of the window remains quite stable at 25°C (changes < 3%) and 40°C (changes < 5%) for a trial period of 10 days (Figure S5). A heating-cooling test of 50 cycles also indicates the stability of the HNAH for long-term operation, which shows less than a 1.5% change in transmittance (Figure 3(f)). It is worthwhile to note that, during the phase transition process, there is no noticeable volume shrinkage (Figure S6), which is another advantage over previous thermochromic hydrogels with volume phase transition, e.g., poly (N-isopropyl acrylamide) (PNIPAM) [32, 33].

In addition to the trivalent aluminium cation, other types of cations, such as monovalent sodium ion and divalent calcium ion, can tune the hydrophilic-hydrophobic balance and render the hydroglass thermochromic in the visible spectrum (Figures S7 and S8). The phenomenon is similar to that of the trivalent cation, that is, the increase of hydrophilicity improves the phase transition temperature and vice versa. But compared to the trivalent cation, the low-valent cations have poorer capacity in the ionic association with the carboxylate, and the tunable phase transition temperature ranges of the HNAH are narrower.

Besides, the monomer concentration also influences the liquid-liquid phase separation of the precursor and hydrophilic-hydrophobic balance of the HNAH. Higher monomer concentration promotes the local enrichment of the polymer chains. Although the phase transition behavior is retained, the transmittance in the visible region declines at room temperature (Figure S9). Hydroglasses with lower monomer concentration, on the other hand, fail to form physically crosslinked networks because of less polymer aggregation domains.

### 2.4. Molecular Mechanism and Multiscale Structural Evolution Analysis

**Figure 4:**
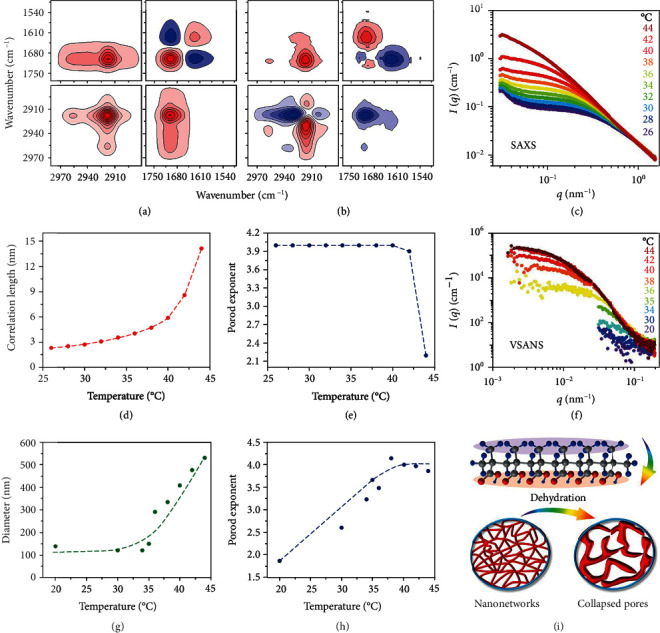
Molecular mechanism and multiscale structural evolution analysis. (a) 2D synchronous spectra of the HNAH during the phase transition. (b) 2D asynchronous spectra of the HNAH during the phase transition. Warm red colors are defined as positive intensities, while cool blue colors suggest negative ones. (c) Temperature-dependent SAXS curves of the HNAH. (d) The correlation length vs. temperature curve of the HNAH derived from the SAXS result. (e) The porod exponent plot derived from the SAXS result. (f) Temperature-dependent VSANS curves of the HNAH. (g) The diameter vs. temperature curve of the HNAH derived from the VSANS result. (h) The porod exponent plot derived from the VSANS result. (i) Schematic models of the molecular response and nanonetwork revolution during the phase transition.

The molecular mechanisms and multiscale structural evolution behind the dynamic response of the HNAH are further analyzed by two-dimensional correlation spectroscopy (2Dcos), temperature-dependent SAXS, and VSANS (Figure 4). The HNAH with about 550 mM positive charge concentration of the trivalent aluminium cation is taken as an example. First of all, different molecular motions, including hydrophobic *α*-methyl and methylene structures and hydrophilic carboxylic acid groups, are tracked by 2Dcos to provide insights into molecular interactions behind the structural evolution (Figure 4(a) and (b), Figure S10 and more details are available in Note S3) [17, 34]. The bands located at 1750-1670 cm^−1^ and 2970-2900 cm^−1^ belong to *v*(C=O) of the carboxylic acid group and *v*(C-H) of the hydrophobic *α*-methyl/methylene structure, respectively. On the basis of 2D synchronous and asynchronous spectra, the sequence order of the stretching modes of different hydrophobic/hydrophilic groups during the phase transition is determined as *ν*(CH_3_) (2957 cm^−1^) → *ν*(CH_2_) (2935 cm^−1^) → *ν*(associated COOH) (1700 cm^−1^) → *ν*(disassociated COOH) (1724 cm^−1^) (Figure 4(b)). It suggests that the pendant *α*-methyl groups firstly respond to the temperature perturbation and then drive the motions of the polymer main chains, as indicated by the methylene group. Finally, these hydrophobic groups promote the disassociation of the hydrogen bonding among the carboxylic acid groups.

SAXS results reveal the evolution of delicate structures below 100 nm. As shown in Figure 4(c), the scattering intensities were fitted by the correlation length model (more details are discussed in Note S4). At the high *q* regime, the correlation length *ξ*, which implies the thickness of the nanonetwork skeletons, increases from several nanometers to a dozen nanometers. It only interferes with the transmission of UV light probably due to Rayleigh scattering but has no effect on visible light, so the hydroglass before the phase transition shows high transmission in visible band. As the temperature increases from 26°C to 44°C, the low *q* intensity becomes stronger after the phase transition, pointing to the formation and growth of the clusters. The power-law exponent *n* decreases, indicating a reduction in the chain-chain interaction. With the 2Dcos results, it suggests that disassociation of the hydrogen bonding among the carboxylic acid groups directly leads to the collapse of nanonetworks. On the other hand, VSANS results show the evolution of the structures located at the range of 15-2000 nm. The diameters of the nanonetworks, which are calculated from *R*_g_, increases from about 100 nm at room temperature to >290 nm above 36°C. The porod exponent increases from 1.86 to 4, indicating the aggregation and merging of nanonetworks of around 100 nm. Meanwhile, the generation of dense collapsed structures with the larger pore sizes of at least several hundreds of nanometers probably results in very strong scattering (Mie scattering) in the visible band and thus decreases the transmittance after the phase transition.

Considering the 2Dcos, SAXS, and VSANS results, the hydrophobic *α*-methyl group plays a key role to initiate the phase transition. As illustrated schematically in Figure 4(e), the *α*-methyl group is supposed to drive the dehydration of the polymer main chains, cause the initially flexible chains to become rigid in water, and disassociate the hydrogen bonding among the carboxylic acid groups. Finally, the breaking hydrogen bonding results in a collapsed structure with increasing scattering geometry, and thereby, the visible transmittance is influenced.

### 2.5. UV-Blocking and Biosafety

**Figure 5:**
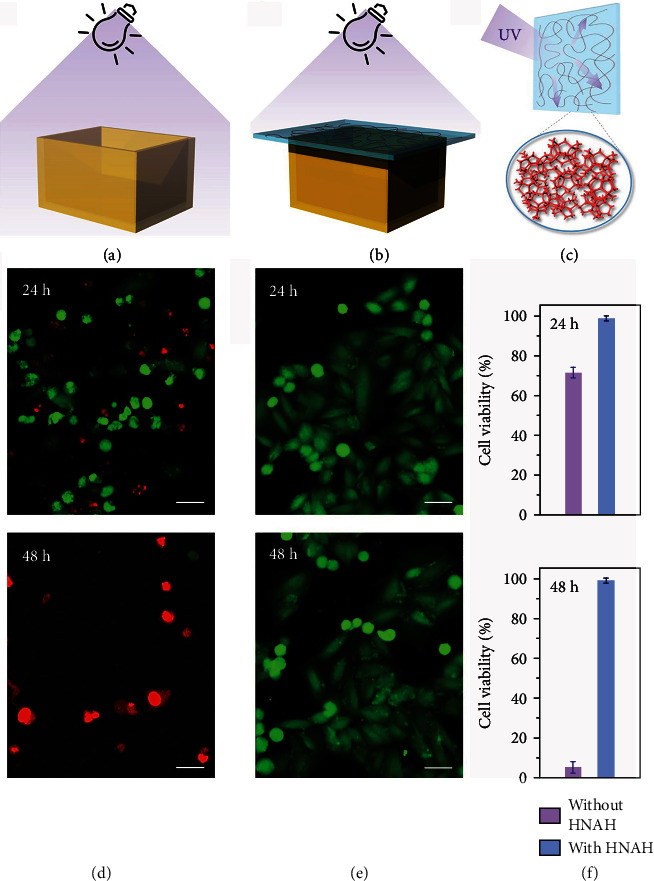
UV-blocking and biosafety of the hydroglass. (a) Schematic illustration of the UV safety test on cell viability without HNAH window protection. (b) Schematic illustration of UV safety test on cell viability with HNAH window protection. (c) Schematic illustration of the scattering effect by the nanonetworks in HANH. (d) The confocal optical imaging of the cells after UV irradiation without HNAH window protection and being further cultured for 24 and 48 h. (e) The confocal optical imaging of the cells after UV irradiation with HNAH window protection and being further cultured for 24 and 48 h. (f) The cell viability after UV irradiation with or without HNAH window protection and being further cultured for 24 and 48 h. Scale bar: 50 um.

In addition to the dynamic light regulation in the visible region, the HNAH extends the light managing ability to UV region. The transmittance of the raw materials (MAA monomer with APS initiator and AlCl_3_ aqueous solutions) shield most of UVC (200-275 nm) and UVB (280-320 nm) probably due to the absorption of their chemical groups, but hardly block the UV light above 300 nm. After polymerization, UV light above 300 nm strongly attenuates (Figure S11). Since the chemical groups in HNAH remain almost the same as the raw materials except for the disappear of carbon-carbon double bonds, the strong attenuation of the UVA light above 300 nm is ascribed to the absorption of the emerging nanostructures in HNAH (Figure S12). This feature is good for biosafety because the UV light is harmful to most living creatures. We monitor the health conditions of living cells with or without the protection of the HNAH window (Figure 5). Under the irradiation for 30 min, the cell viability declines to about 71.4% in the next 24 h and about 5.5% in 48 h. In contrast, with the protection of the HNAH window, the cell viability maintains 99.1% in the next 48 h, similar to the control group without UV irradiation (Figure S13).

### 2.6. Infrared Stealth and Energy Saving

**Figure 6:**
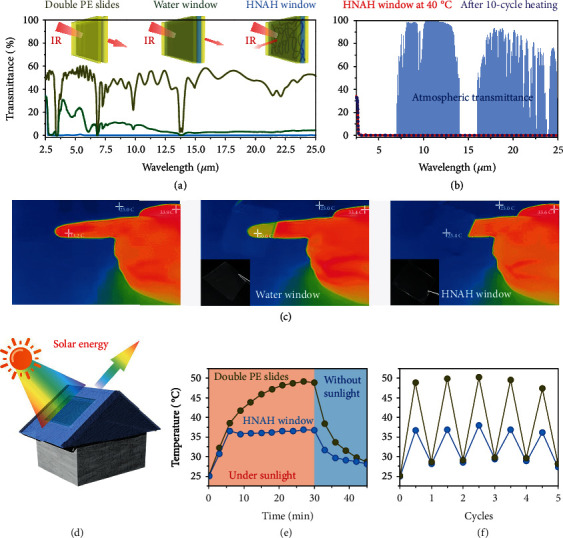
Infrared stealth and energy saving of the hydroglass. (a) The transmittance results of the double PE slides, double PE slides with water interlayer (water window), and HNAH window in the IR band. (b) The IR transmittance results of the HNAH window at 40°C and after 10-cycle heating. (c) The thermal infrared photographs of a naked human hand (left), the hand covered by water window (middle), and the hand covered by the HNAH window (right). The insert photographs are the water window and the HNAH window, respectively. (d) Schematic illustration of a model chamber with different window materials. (e) Temperature profiles of the thermometer inside the model chamber with double PE slides or HNAH as the window materials. (f) Cyclic temperature tests recorded by the thermometer inside the model chamber with different window materials.

We further study the optical characteristics of HNAH in the IR band. A typical polymer, for example, polyethene (PE), is semitransparent in the IR region because some of IR light is absorbed due to the molecular vibration. As shown in Figure 6(a), a PE window with two slides of 45-micron PE is semitransparent in the range of 2.5-25 *μ*m. With the addition of a 50-micron water layer, the transmittance decreases, but this water window is still transparent in the range of 3-12 *μ*m. In contrast, for an HNAH window with a piece of 50-micron HNAH instead, the IR light with the wavelength ranging from 2.5 to 25 *μ*m is almost blocked. Most of the materials, including the traditional inorganic glasses and Plexiglas, although, have some absorption in the IR region due to the molecular vibration, rare materials show such high blocking efficiency with such a small thickness. The excellent light blocking by the HNAH in the IR band probably results from micronetworks whose dimensionless size parameter reaches unity in the IR region [9]. This speculation is in line with the TEM and FESEM observation. In addition, after the phase transition at 40°C, the broadband IR light is also entirely blocked by the HNAH, and cyclic heating-cooling processes have no interference (Figure 6(b)). It indicates that the HNAH successfully achieve the decoupling modulation of the IR and visible light in the whole solar spectrum.

The broadband IR blocking effect endows the HNAH window with a massive potential in the applications of military stealth and energy saving. For an object with a surface temperature higher than absolute zero, it emits thermal radiation, and the radiation wavelength is located in the IR band, mostly 8-13 *μ*m, which is in the atmospheric transmission window [35, 36]. Therefore, the IR radiation can pass through the atmosphere and be detected by thermal IR cameras [37]. As shown in Figure 6(c), a human hand can be detected with obviously higher temperature (about 35°C) compared to the environmental temperature (about 25°C). In comparison, when we place the visible transparent HNAH window in front of a human finger, it entirely blocks the IR light and makes the finger stealth from the view of the IR camera. However, the water window without the hierarchical networks fails to hide the thermal image of the finger.

Furthermore, the HNAH window is installed on a thermal insulation chamber to study the energy-saving effect. The chamber and an inside thermometer are illuminated by summer sunlight (Figure 6(d)). The initial temperature recorded by the thermometer is about 25°C when the chamber is just taken from an air-conditioned room and increases to 36.5°C under sunlight illumination for 6 min (Figure 6(e)). The HNAH window spontaneously switches from transparent to opaque states. After 18 min of illumination, the inside thermometer maintains a plateau temperature of about 36°C. Double PE slides are used for a control experiment. The thermometer under the PE window shows much higher heating rate with sunlight illumination. The temperature quickly increases to 38.6°C after 6 min. The plateau temperature is about 49°C, which is 13°C higher than that of under the HNAH window. Such impressive cooling effect is ascribed to the reason that the HNAH window simultaneously isolates visible light and IR light and decreases the solar energy absorbed by the inner thermometer. When stopping the sunlight illumination, the temperature recorded by the thermometer under the HNAH window decrease to 28.1°C in 15 min. Furthermore, cyclic tests are performed from 11 am to 3 pm. The results confirm the stable solar energy shielding behavior of the HNAH window (Figure 6(f)).

## 3. Discussion

In this work, we develop the next-generation versatile glass named HNAH with hierarchical networks via LCP approach. It is highly transparent in the visible region at room temperature and can manage the light wavelength over three orders of magnitude. The HNAH with hierarchical networks ranging from nano- to micro-scale is significantly distinct from conventional transparent glasses with homogenous networks at the nanoscale. It not only realizes the broadband light management but also shows enhanced mechanical properties with elasticity, notch resistance, and self-healing ability. As a smart window, the hierarchical networks can block light in the harmful UV band and shield the solar energy from both visible and IR regions. A decline of the indoor temperature of 13°C is expected with the installation of HNAH.

Moreover, the molecular interactions and structural evolutions during the LCP and light management are discussed. The key point is the amphiphilicity, especially the *α*-methyl group, which not only facilitates the LCP but also promotes the structural evolution during the phase transition. We believe the LCP strategy and the molecular mechanisms may also apply to other light management materials, from organic liquid crystals to inorganic oxides. The analogous smart windows that can modulate light in a broad band ranging from UV to IR region promise to be good for human health, reduce building energy consumption, and address environmental issues.

## 4. Materials and Methods

### 4.1. Materials

MAA, AlCl_3_, CaCl_2_, NaCl,_3_)_3__2_, ammonium persulfate (APS), N, N-methylenebisacrylamide (BIS), and D_2_O were purchased from Sigma-Aldrich Co. Cell Imaging Kit (LIVE/DEAD™ Cell Imaging Kit (488/570)) was obtained from ThermoFisher. Dulbecco's modified Eagle medium (DMEM) was purchased from Gibco. Phosphate-buffered saline solution (PBS, pH 7.4) and antibiotics (100 units·mL^−1^ streptomycins and 100 *μ*g·mL^−1^ penicillin) were purchased from Invitrogen.

### 4.2. Preparation of the HNAH

HNAH was prepared by a one-step radical polymerization of MAA monomers in the absence of crosslinkers. The monomer concentration was kept at 20 wt% unless noted otherwise. The salts were added in the precursor monomer solutions, and the polymerization was initiated by 0.2 mol% APS and proceeded at 70°C for 6 h. The phase transition behavior of the HNAH was tunable based on the different amount of salts.

### 4.3. Cell Culture and Damage Assessment

Hela cells were seeded on 96-well plates (2 × 10^4^ cells/well) and cultured in DMEM with 10% PBS plus 1% penicillin/streptomycin. The plates were firstly incubated in a humidified 5% CO_2_ incubator at 37°C for 12 h. Then, the medium was replaced with fresh growth medium. The experimental groups were exposed under UV light (254 nm) with or without the protection of the HNAH window. After 30 min exposure, the cells are further cultured for another 24 h or 48 h for live/dead imaging assay. The control group was cultured without UV irradiation.

### 4.4. Characterization

Transmission electron microscope (TEM) images were observed by a JEOL JEM2011 at 200 kV. FESEM image was recorded by Zeiss Ultra 55. The optical micrographs of the polymerization process of the hydroglass were observed by an optical microscope (Leica DM2500P). The UV-Vis spectra were collected on a Lamda 35 spectrophotometer. The temperature-dependent transmittance spectra were recorded at 550 nm with water as the reference. The hydroglasses were sealed in a quartz cell. Because of the limit of the low thermal conductivity for aqueous polymers, which is typically <1 W m^−1^ K^−1^, the actual heating rate of the hydrogalss cannot match very high heating rate. We choose the heating rate of 1°C min^−1^ unless otherwise stated. Temperatures were controlled automatically with water-jacketed cell holder. In order to reach the thermal equilibrium, each temperature point was stabilized for 120 s before measurement. The heating-cooling test of 50 cycles was conducted at the temperatures of between 25 and 40°C. The reflectance spectrum was measured using a UV-VIS spectrometer (UV2600, Shimadzu) with a diffuse integrating sphere. The absorbance spectrum was calculated from the relationship of *ε*(*λ*) = 100% − *τ*(*λ*) − *ρ*(*λ*), in which the *ε*(*λ*), *τ*(*λ*), and *ρ*(*λ*) are the absorbance, reflectance, and transmittance of the sample at the wavelength *λ*. The IR spectra of the HNAH were collected on a Nicolet 6700 spectrometer using transmittance method. Confocal imaging was performed on a Leica TCS-SP5 confocal laser scanning microscope. The infrared thermal images with IR transmittance information were captured by a FLIR One Pro camera. For the chamber temperature measurement, two thermal insulation foam boxes were inserted with thermocouples. One installed the HNAH window, and another installed a PE window with the same size. When they were irradiated under the sunlight, real-time temperatures were recorded by the thermocouples.

ATR spectra were recorded by using a diamond crystal as the window material. Time-resolved of the LCP process were collected every 2 min. Temperature-dependent ATR spectra were recorded with a temperature interval of 1°C. The hydroglasses are pressed at the ATR window and sealed by tetrafluoroethylene tapes and parafilm. The software of 2D Shige ver. 1.3 developed by Shigeaki Morita (Kwansei Gakuin University) was used for the 2Dcos analysis. In the contour maps, red colors indicate positive intensities, and the blue colors indicate negative ones.

Tensile curves were recorded on a universal mechanical test machine (Instron 5966). The tensile stress-strain curve of the self-healing sample was recorded from a completely fractured sample which was self-healing for 48 h. DIC, as a noncontact optical technique, was applied for the full-field strain measurement and the corresponding stress imaging on a notched HNAH [26]. A random speckle pattern was firstly spray-painted on the notched HNAH surface. The initial crack length of the notched sample is around 1 mm. The notched sample was cyclically stretched between 0 and 100% deformation strain. Images throughout cyclic extension were recorded by a standard video camera on the Instron 5966. The surface strain mapping was analyzed by VIC-2D software.

SAXS experiments were performed at the SSRF beamline BL16B (Shanghai, P.R. China) at an X-ray energy of 10.0 keV which corresponds to a wavelength of *λ* = 1.24 Å. The sample-detector distances of 1.98 m and 5 m cover the scattering vector *q* range from 0.03 to 4.5 nm^−1^ (*q* is the scattering vector, *q* = (4*π*/*λ*)sin(*θ*), 2*θ* is the scattering angle). Samples were measured in a liquid cell in a thermal stage. The scattering patterns were obtained with short exposure time (10 s to 180 s). The SAXS patterns were normalized to an absolute scale and azimuthally averaged to obtain the intensity profiles, and the solvent background was subtracted. For SAXS experiments on Gel/sol samples measured in a liquid cell with an internal diameter of 1 mm, water and solvent are also measured in the same capillary with the same instrument configuration. The final scattering intensity of the sample is

(1) $$\begin{array}{c} I\left(q\right)=\frac{\left(I{\left(q\right)}_{s}/t_{s}T_{s}\right)-\left(I{\left(q\right)}_{b}/t_{b}T_{b}\right)}{{\left(\left(I_{w}/t_{w}T_{w}\right)-\left(I_{EC}/t_{EC}T_{EC}\right)\right)}_{1{nm}^{-1}\leq q\leq 4{nm}^{-1}}}*0.0164. \end{array}$$

In Equation (1), *I*(*q*)_s_, *I*(*q*)_b_, *I*(*q*)_w_, and *I*(*q*)_EC_ are the measured intensity of sample, solution background, water, and empty cell, respectively. The bottom term in the high *q* range is a flat scattering (*q* independent), whereas *T* is the transmission and *t* the irradiation time. The approach used to evaluate the SAXS data applies a set of general laws (Porod's law, Guinier analysis) that yield results right after data reduction. Then, the scattering curves were fitted with models that yield accuracy parameters for data analysis.

VSANS experiments were carried out at the KWS-3 diffractometer using a parabolic mirror as an optical element and covering the smaller *Q* range from 0.0006 to 0.25 nm^−1^. VSANS experiments were carried out at KWS-3 diffractometers operated by the Jülich Centre for Neutron Science (JCNS) at the Heinz Maier-Leibnitz Zentrum (MLZ) in Garching, Germany. Two configurations were used at KWS-3, namely, the sample-to-detector (SD) distances of 1.25, and 9.3 m, and wavelength of 12.8 Å (Δ*λ*/*λ* = 17%). These settings allowed covering a *Q* range from 0.001 to 0.25 nm^−1^. A two-dimensional position sensitive detector was used to detect neutrons scattered from the samples. The hydroglasses were filled into a copper cell with two quartz windows and path-length of 1 mm. The CoolerHeater sample stage uses a Peltier element to heat and cool samples. Temperature can be continuously controlled from -20°C to +140°C. Direct beam method was used at KWS-3. The data correction and calibration were performed using the software QtiKWS. Fitting the SANS/VSANS data was done using software modules provided by NIST Igor analysis packages.

## Data Availability

The data that support the findings of this study are available from the corresponding author upon reasonable request.

## Supplementary Materials

Supplementary Materials Note S1: time-resolved ATR spectra of the LCP process. Note S2: apparent activation energy *E*_a_ of the hierarchical networks. Note S3: introduction of two-dimensional correlation spectroscopy (2Dcos). Note S4: theory models for SAXS and VSANS. Figure S1: the photograph of the MAA aqueous solution. Figure S2: UV-Vis spectrum of the HNAH. Figure S3: the FESEM image of the freezing-drying HNAH. Figure S4: heating rate dependent turbidity results. Figure S5: relative weight of the HNAH at different temperatures for a long time. Figure S6: photographs of the HNAH before and after phase transition. Figure S7: transmittance vs. temperature for the HNAH with different cation concentrations (NaCl). Figure S8: transmittance vs. temperature for the HNAH with different cation concentrations (CaCl_2_). Figure S9: transmittance vs. temperature for the HNAH with different monomer concentrations. Figure S10: temperature-dependent FTIR results of the HNAH. Figure S11: UV-Vis transmittance spectra of the AlCl_3_ aqueous solution, monomer aqueous solution, and the HNAH. Figure S12: UV-Vis reflectance and absorbance spectra of HNAH. Figure S13: the confocal optical imaging and the cell viability of the cells in the control group.
