# Supplementary material for: Hierarchical Network-Augmented Hydroglasses for Broadband Light Management
Source: Research (Wash D C). 2021 Jan 20;2021:4515164. doi: 10.34133/2021/4515164 (PMC7877396; doi:10.34133/2021/4515164)
Supplement: Supplementary Materials — Note S1: time-resolved ATR spectra of the LCP process. Note S2: apparent activation energy Ea of the hierarchical networks. Note S3: introduction of two-dimensional correlation spectroscopy (2Dcos). Note S4: theory models for SAXS and VSANS. Figure S1: the photograph of the MAA aqueous solution. Figure S2: UV-Vis spectrum of the HNAH. Figure S3: the FESEM image of the freezing-drying HNAH. Figure S4: heating rate dependent turbidity results. Figure S5: relative weight of the HNAH at different temperatures for a long time. Figure S6: photographs of the HNAH before and after phase transition. Figure S7: transmittance vs. temperature for the HNAH with different cation concentrations (NaCl). Figure S8: transmittance vs. temperature for the HNAH with different cation concentrations (CaCl2). Figure S9: transmittance vs. temperature for the HNAH with different monomer concentrations. Figure S10: temperature-dependent FTIR results of the HNAH. Figure S11: UV-Vis transmittance spectra of the AlCl3 aqueous solution, monomer aqueous solution, and the HNAH. Figure S12: UV-Vis reflectance and absorbance spectra of HNAH. Figure S13: the confocal optical imaging and the cell viability of the cells in the control group. [file 4515164.f1.docx]

Supplementary Materials

Note S1. Time-resolved ATR spectra of the LCP process. Note S2. Apparent activation energy *E*_a_ of the hierarchical networks. Note S3. Introduction of Two-Dimensional Correlation Spectroscopy (2Dcos). Note S4. Theory models for SAXS and VSANS. Figure S1. The photograph of the MAA aqueous solution. Figure S2. UV-Vis spectrum of the HNAH. Figure S3. The FESEM image of the freezing-drying HNAH. Figure S4. Heating rate dependent turbidity results. Figure S5. Relative weight of the HNAH at different temperatures for a long time. Figure S6. Photographs of the HNAH before and after phase transition. Figure S7. Transmittance vs. temperature for the HNAH with different cation concentrations (NaCl). Figure S8. Transmittance vs. temperature for the HNAH with different cation concentrations (CaCl_2_). Figure S9. Transmittance vs. temperature for the HNAH with different monomer concentrations. Figure S10. Temperature-dependent FTIR results of the HNAH. Figure S11. UV-Vis transmittance spectra of the AlCl_3_ aqueous solution, monomer aqueous solution and the HNAH. Figure S12. UV-Vis reflectance and absorbance spectra of HNAH. Figure S13. The confocal optical imaging and the cell viability of the cells in the control group.

**Note S1. Time-resolved ATR spectra of the LCP process**

We drop the precursor solution with the liquid-liquid phase separation phenomenon on the ATR diamond window and initiate in-situ polymerization with the irradiation of an ultraviolet lamp (365 nm). Time-resolved ATR spectra are recorded for analyzing the molecular motions and interactions during the LCP process. Note that in order to eliminate the overlap of H_2_O at around 1640 cm^-1^, D_2_O instead of H_2_O is used as the solvent. Besides, in order to eliminate the influence of temperature variables during the thermally initiated polymerization, AIBA initiator instead of APS is used for the in-situ polymerization at room temperature. The characteristic peak located at 1630 cm^-1^ corresponds to the carbon-carbon double bond of the monomer. When the reaction proceeds to 16 minutes, this characteristic peak almost disappears, indicating that the LCP is completed. During this process, we observe that both *ν*(CH_3_) and *ν*(CH_2_) shift to higher wavenumbers, indicating that the originally hydrophobic groups, α-methyl and methylene, are gradually hydrated during the LCP process, and the resulting polymer is more hydrophilic than the monomer. Therefore, the liquid-liquid phase separation gradually disappears during the LCP process.

**Note S2. Apparent activation energy *E*_a_ of the hierarchical networks**

The apparent activation energy *E*_a_ is obtained from the Arrhenius equation,

$a_{T} ={Ae}^{E_{a}/RT}$ (1)

in which $a_{T}$ is the horizontal shift factor, R is the ideal gas constant, and A is a constant.([*1*](#_ENREF_1)) The apparent activation energy value (149 kJ mol^-1^) is calculated from the slope of the curve in Figure 2b.

**Note S3. Introduction of Two-Dimensional Correlation Spectroscopy (2Dcos)**

Two-dimensional correlation infrared (IR) spectroscopy was proposed by Noda in 1986 and further extended to generalized two-dimensional correlation spectroscopy (2Dcos). Compared with traditional 1D IR spectra and temperature-dependent IR spectra, 2Dcos can provide more details on molecular interactions and especially the sequence order of characteristic groups for nearly any kinds of stimuli-responsive systems, for example, time-dependent properties, thermo/photo-responsive behaviors, dynamic mechanical performance and even catalytic mechanisms.([*2-6*](#_ENREF_2)) It is due to the fact that, with an additional dimension, 2D spectra introduce Fourier transforms to resolve the spectral features. Furthermore, the integration of 2D spectra of individual Fourier components results in the final overall synchronous and asynchronous spectra.([*2*](#_ENREF_2)*,* [*7*](#_ENREF_7)) Therefore, 2Dcos not only shows significantly enhanced spectral resolution and thus can distinguish complex responsive behaviors at the molecular level, but also provides comprehensive information of different dynamic interactions, *i.e.*, *via* the sequential order of spectral changes. On the one hand, the synchronous spectra reflect synergistic changes between two wavenumbers; on the other hand, the asynchronous spectra mainly enhance the spectral resolution. In the 2Dcos analysis, synchronous spectra reflect the relative degree of in-phase response between two given wavenumbers while asynchronous one illustrates out-phase response similarly. According to Noda’s rule: when the cross-peaks of *v*_1_ and *v*_2_ (*v*_1_>*v*_2_) in synchronous and asynchronous spectra have the same symbol (either positive or negative), then the change at peak *ν*_1_ has an earlier response than that at peak *ν*_2_, and vice versa.([*8*](#_ENREF_8))

During the past decades, 2Dcos analysis is demonstrated to be a robust and powerful tool in studying diverse, dynamic systems. In this work, we apply this method to analyze the HNAH and try to figure out the hydrophilic-hydrophobic relationships and the driving force during the phase transition. The comprehensive understanding of such complex and subtle dynamic interactions is difficult to be achieved *via* other methods.

**Note S4. Theory models for SAXS and VSANS**

The Beaucage expression:

The Beaucage empirical expression is able to reasonably approximate the scattering from many different types of particles, including fractal clusters, random coils (Debye equation), ellipsoidal particles, etc.([*9*](#_ENREF_9)) The Beaucage expression is given according to:

 (2)

representing a combination of Guinier’s and Porod’s law describing the scattering at low and large *Q*, respectively. More quantitatively both approximations are valid for the parameter *u = R_g_Q* smaller or larger than 1, *u* representing the product of the radius of gyration *R_g_* and the scattering vector *Q*. Guinier's law has the shape of a Gaussian function whereas, for *Q* larger than 1/*R*_g_ (u>1), a power-law according to (3)

is contained.

The correlation length model:

The scattering from PAAM molecules and their clustering effect are well described by the empirical functional model developed by B. Hammouda et al.([*10*](#_ENREF_10))

 (4)

In the equation (4), a scattering of the polymer network is described by the first term *A/Q^n^* and is qualitatively similar to Porod-like scattering that usually is used to evaluate the clustering strength of the primary scattering objects. Scattering at larger *Q* is expressed by the second term *C/[1 + (Qξ)^m^]* and has been used to characterize the polymer/solvent interaction and chain solvation characteristics. The correlation length *ξ* represents a weighted average of inter-distances between the PAAM molecules. In this paper, it identifies the PAAM morphology and the structure on the nanoscale. The amplitudes of the Porod and Lorentzian terms (*A* and *C*, respectively), and the Porod and Lorentzian scattering exponents (*n* and *m*, respectively) were obtained by a nonlinear least-squares fit of the data.


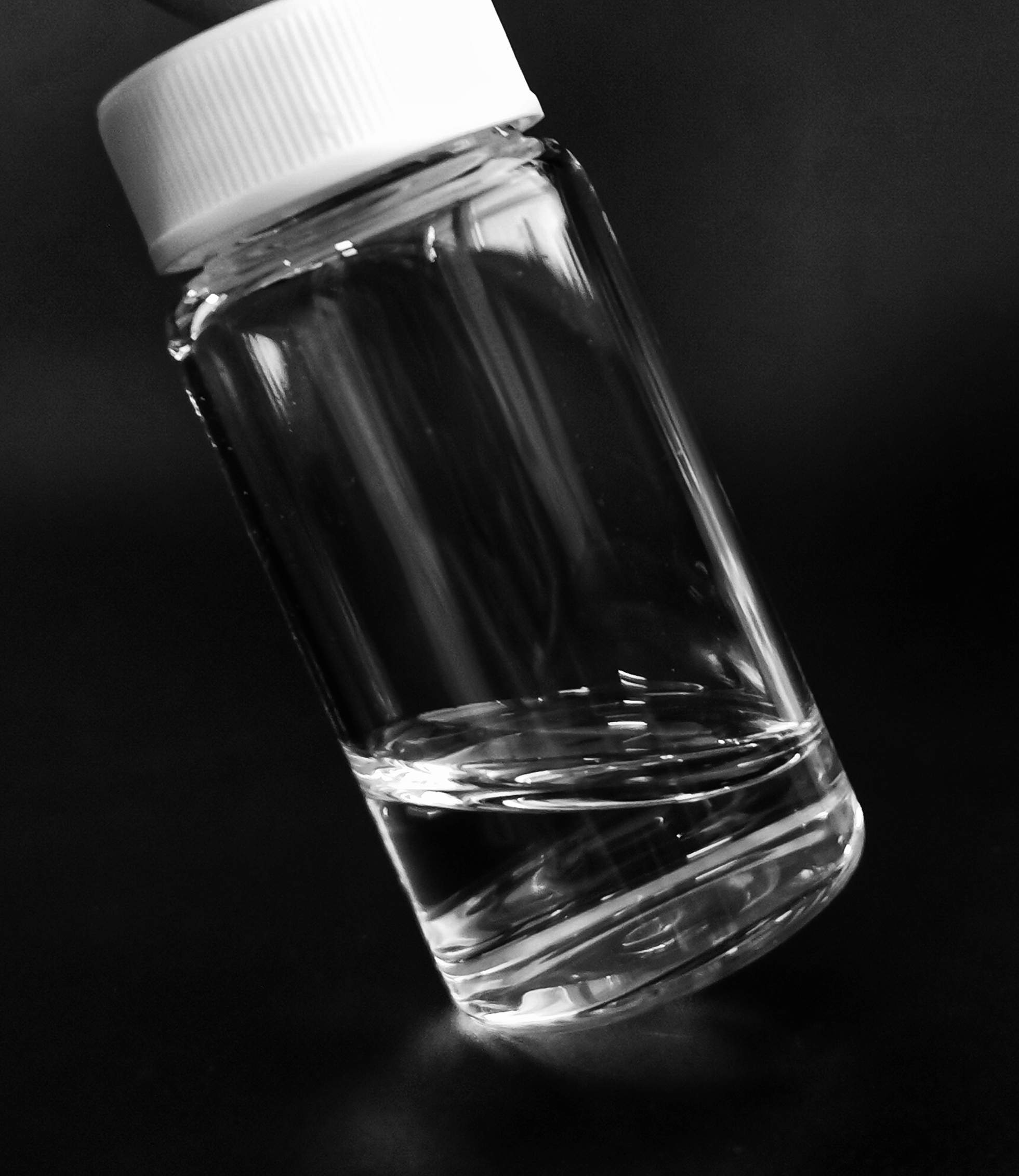


**Figure S1.** The photograph of the MAA aqueous solution without doped cations.


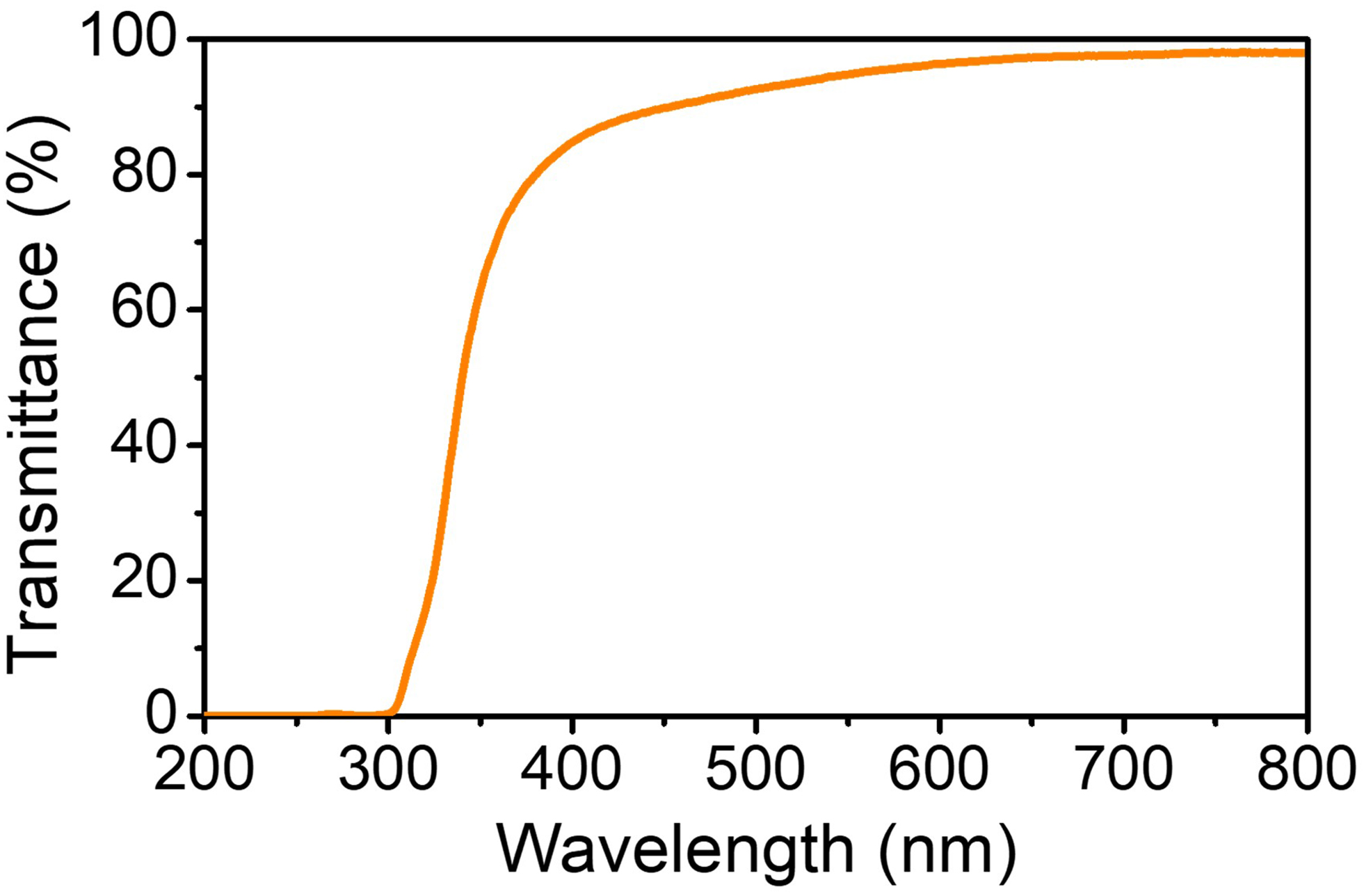


**Figure S2.** UV-Vis spectrum of the HNAH with a thickness of 1 cm.


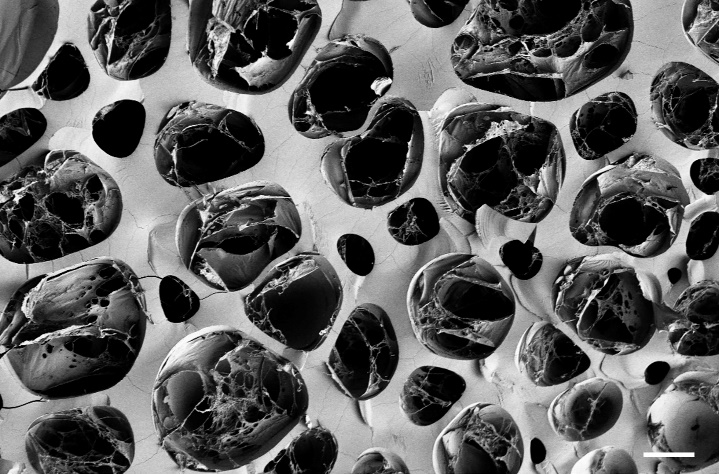


**Figure S3.** The FESEM image of the freezing-drying HNAH. The scale bar is 5 µm.


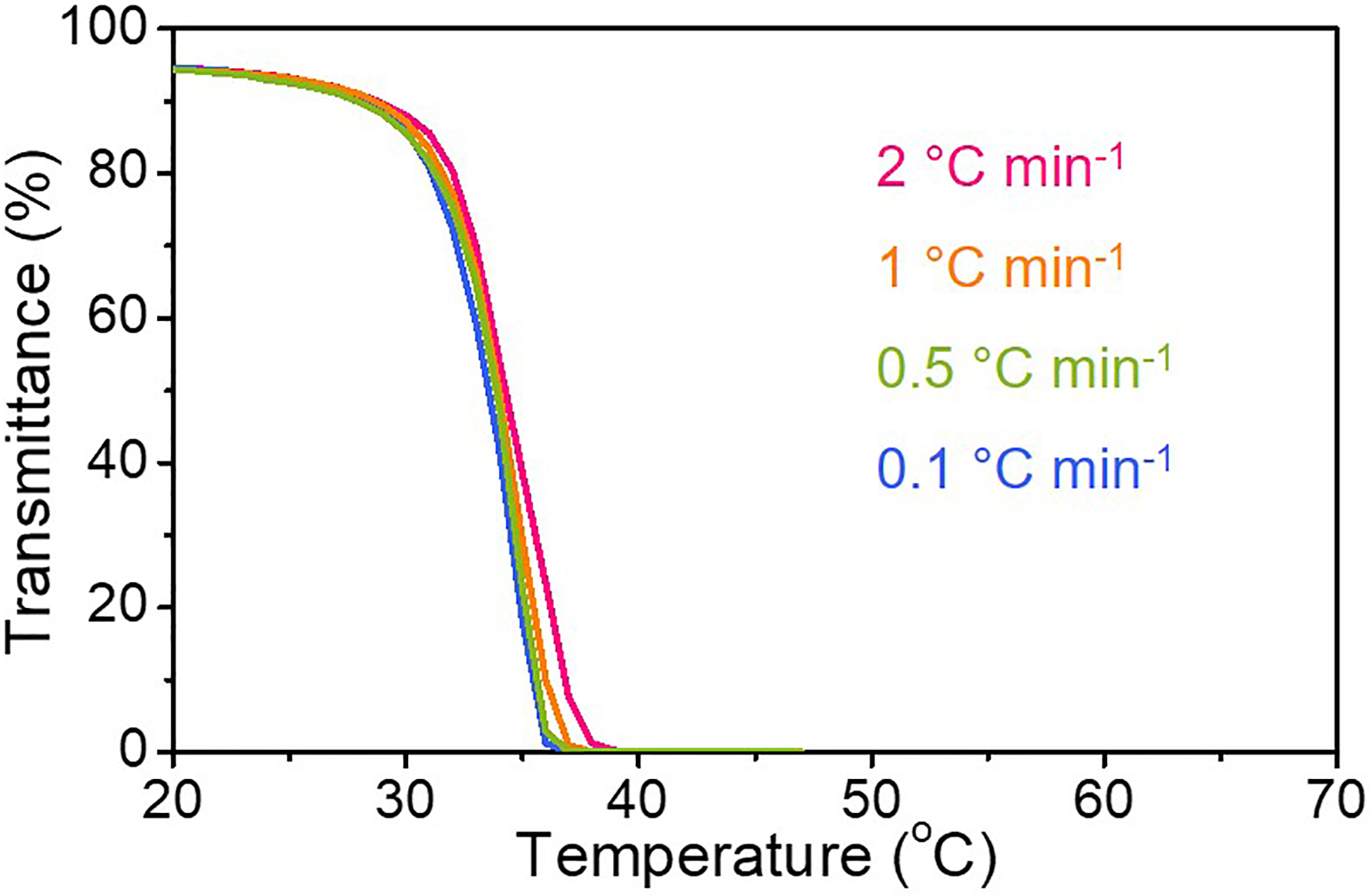


**Figure S4.** Heating rate dependent turbidity measurements.


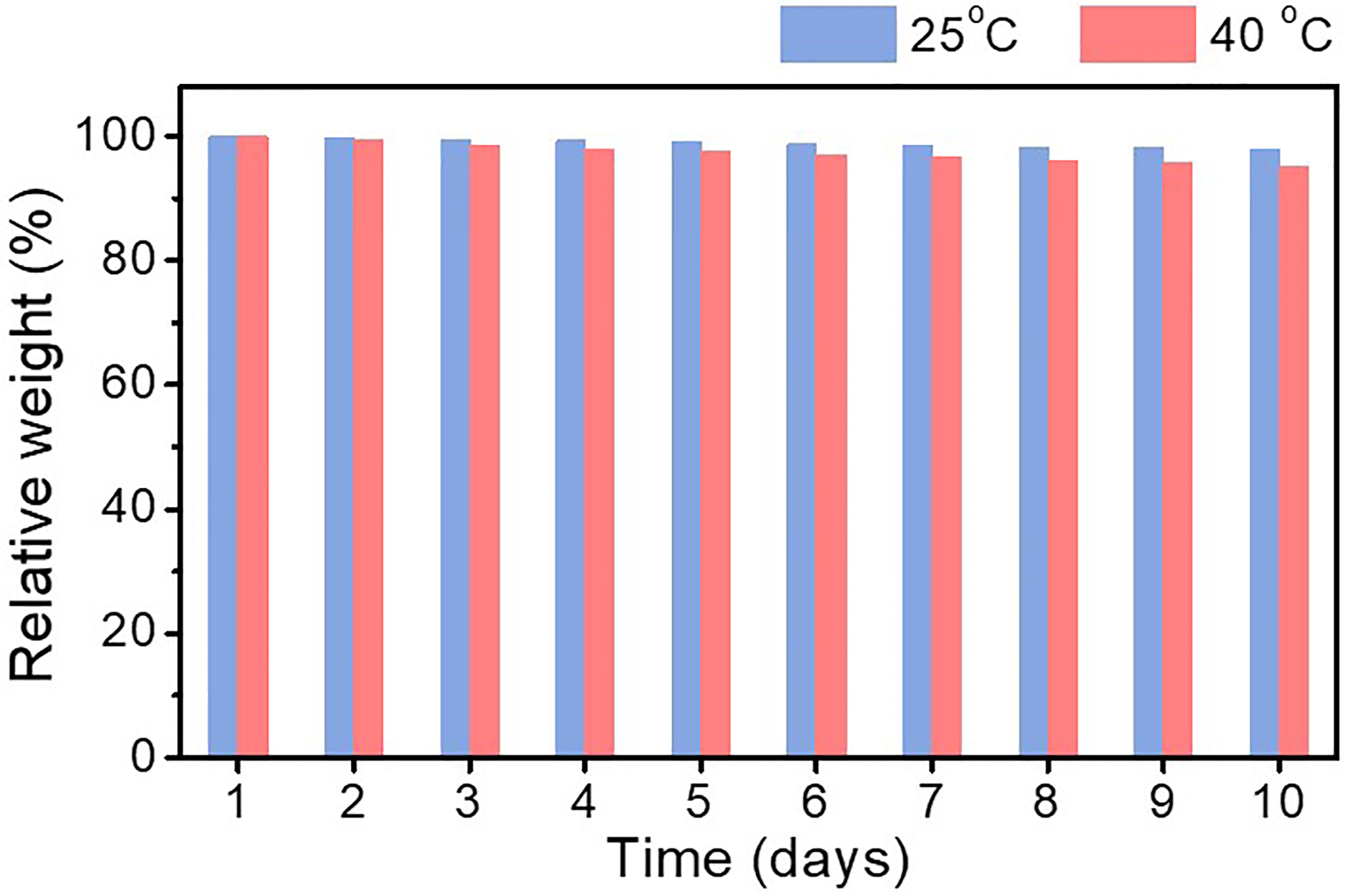


**Figure S5.** Relative weight of the HNAH at different temperatures for a long time.


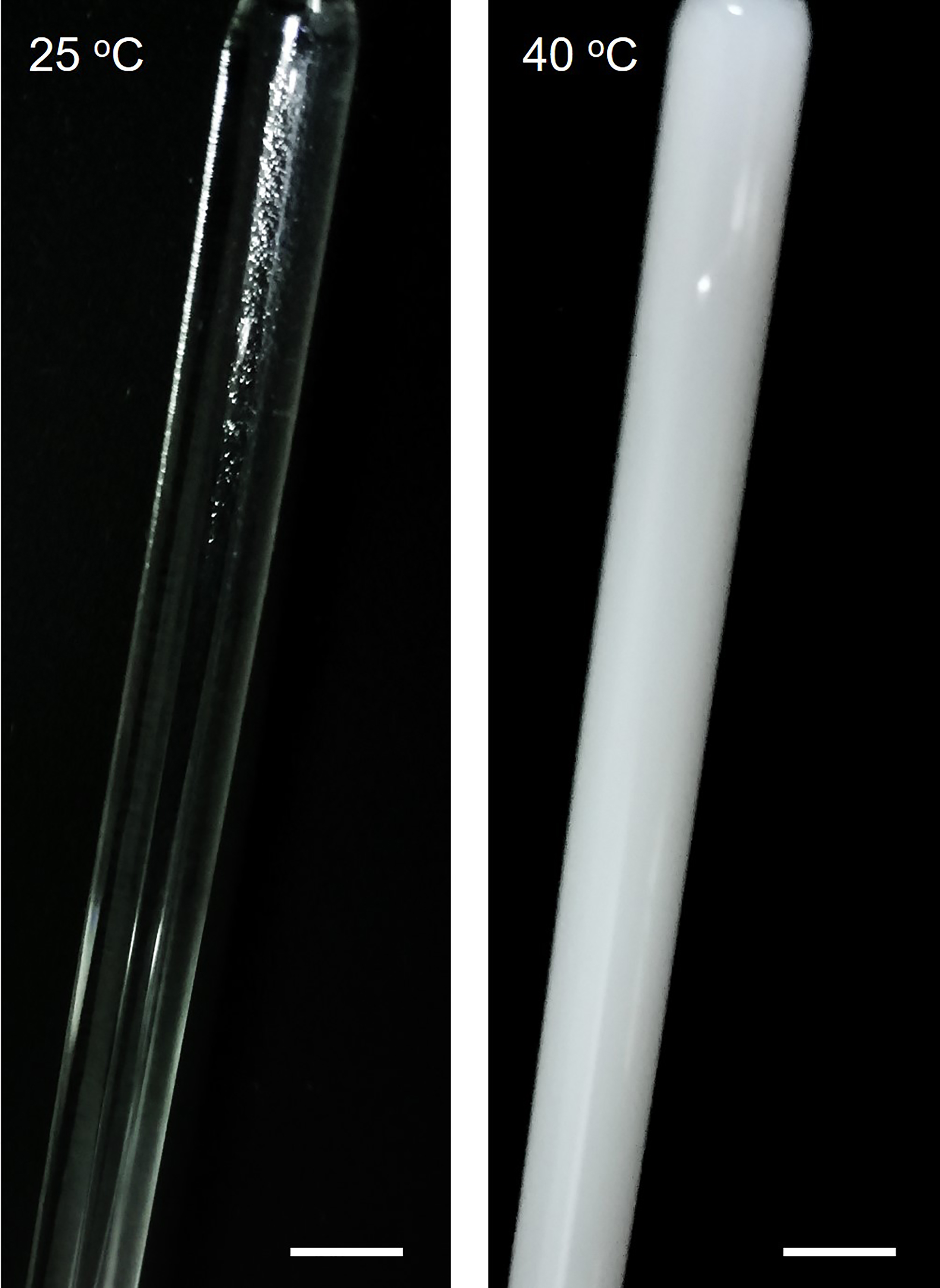


**Figure S6.** Photographs of the HNAH before and after phase transition in a glass tube. They indicate there is no noticeable volume shrinkage after phase transition. The scale bar is 0.5 cm.


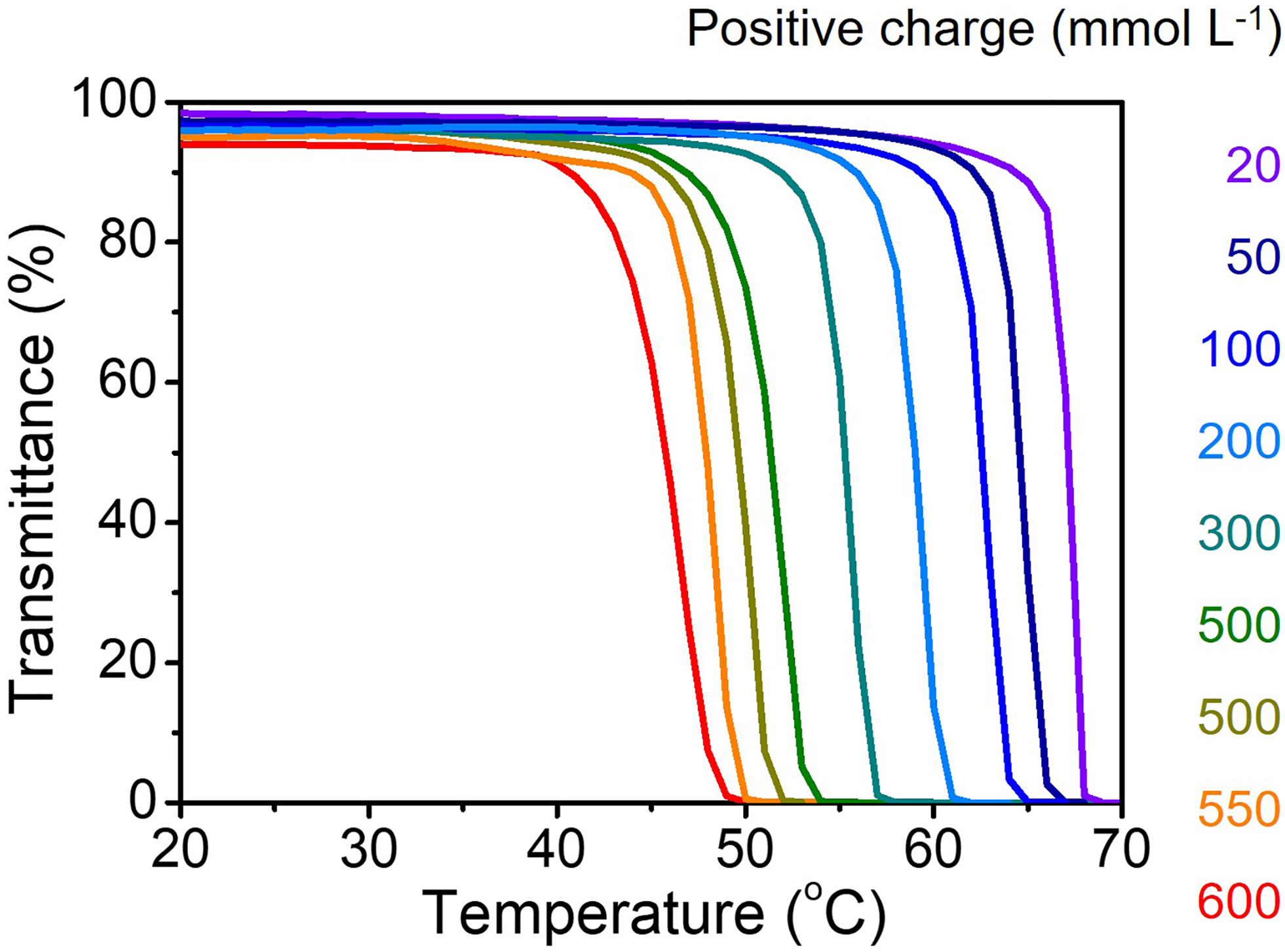


**Figure S7.** Transmittance vs. temperature for the HNAH with different cation concentrations (NaCl).


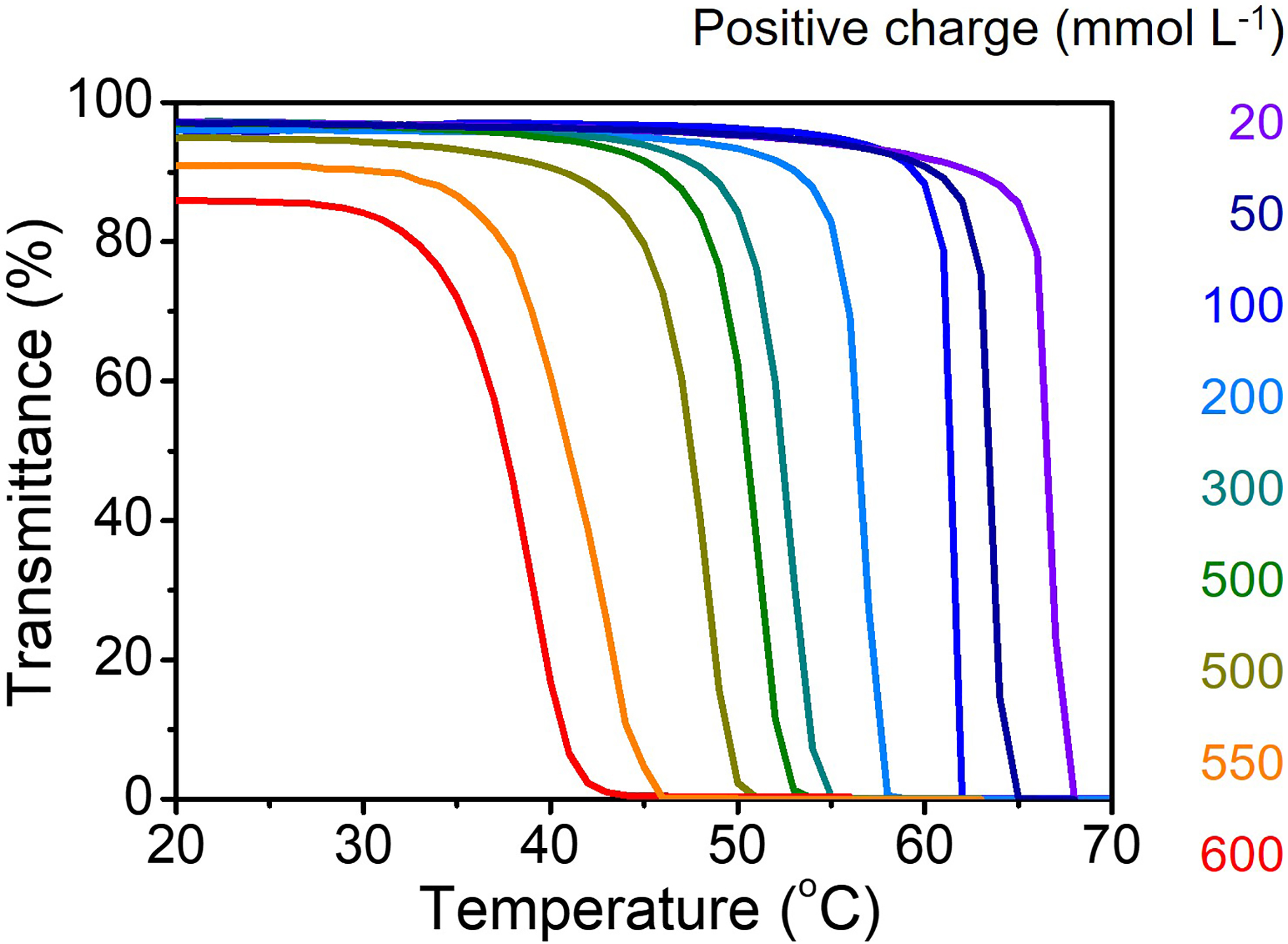


**Figure S8.** Transmittance vs. temperature for the HNAH with different cation concentrations (CaCl_2_).


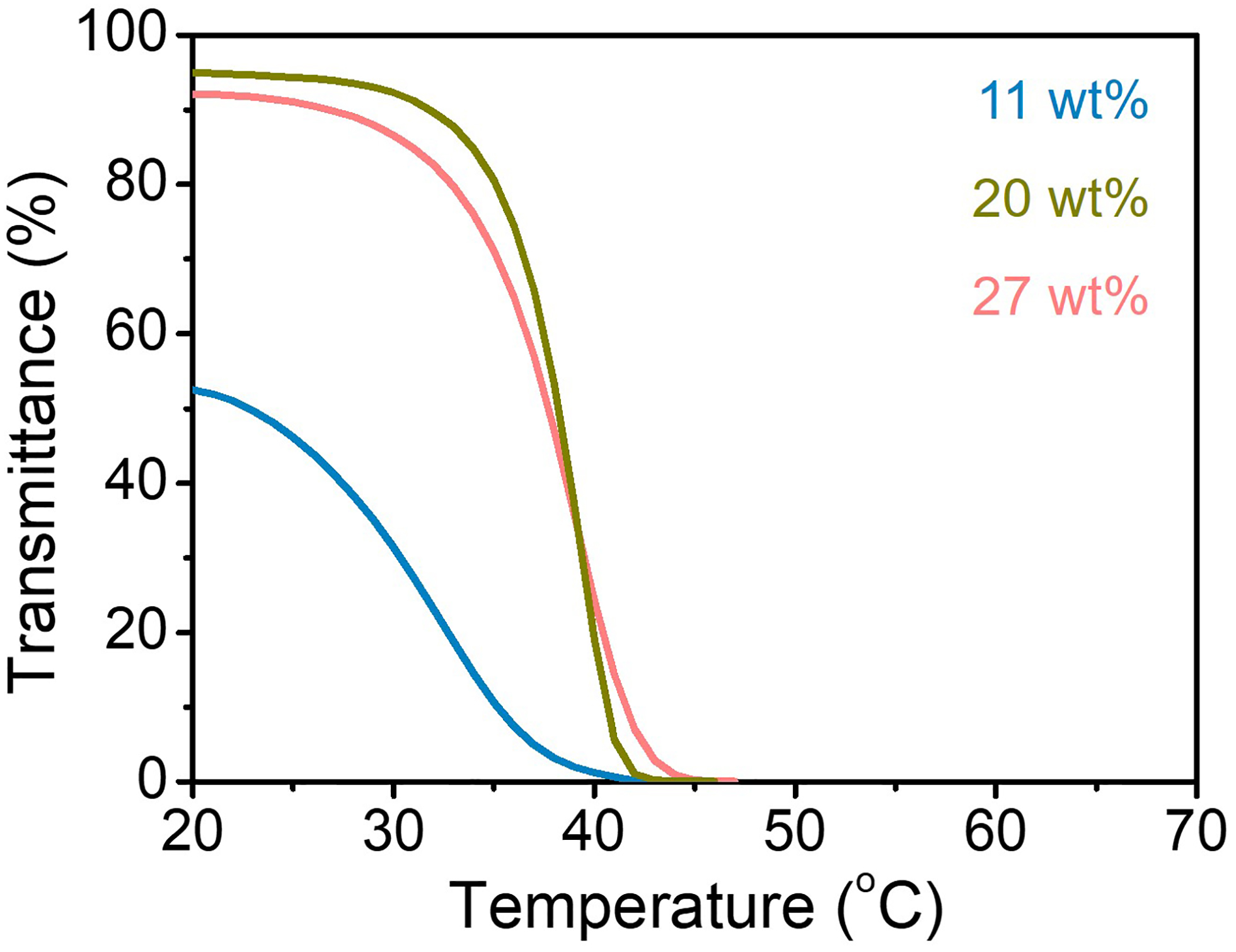


**Figure S9.** Transmittance vs. temperature for the HNAH with different monomer concentrations.


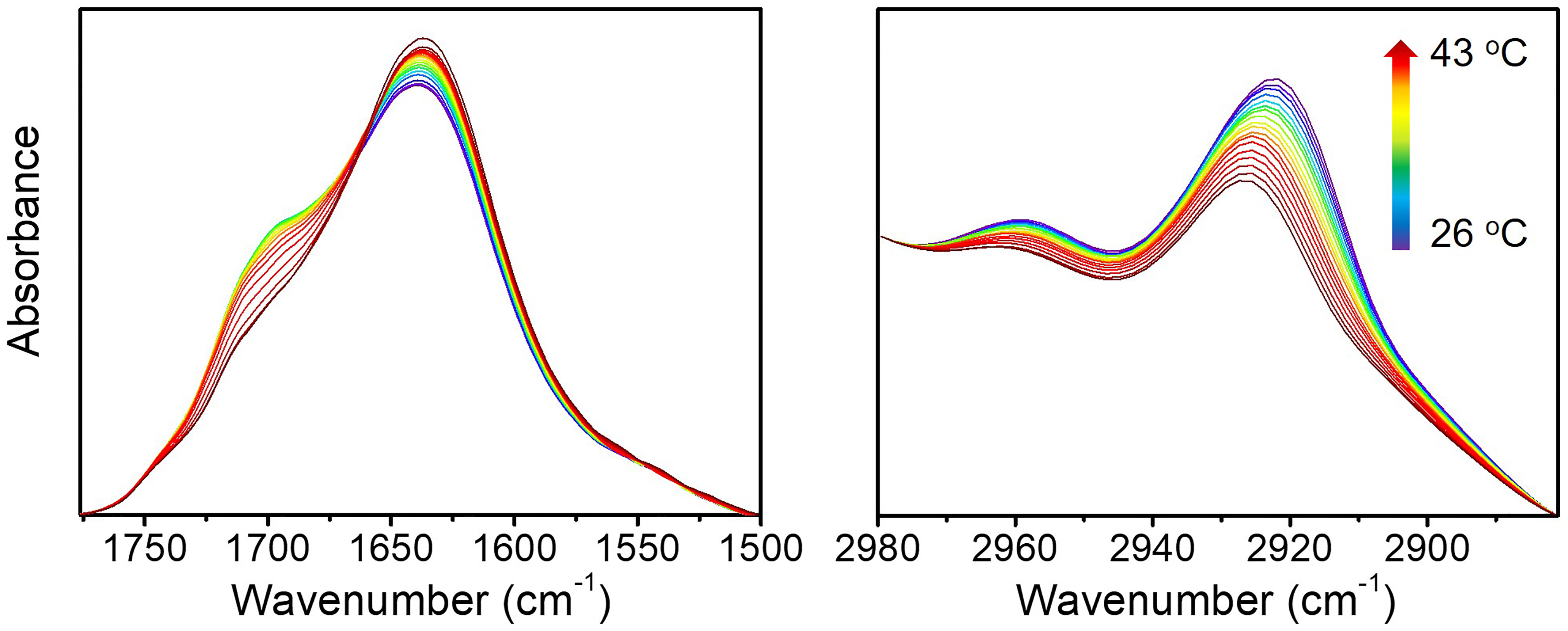


**Figure S10.** Temperature-dependent FTIR results of the HNAH.


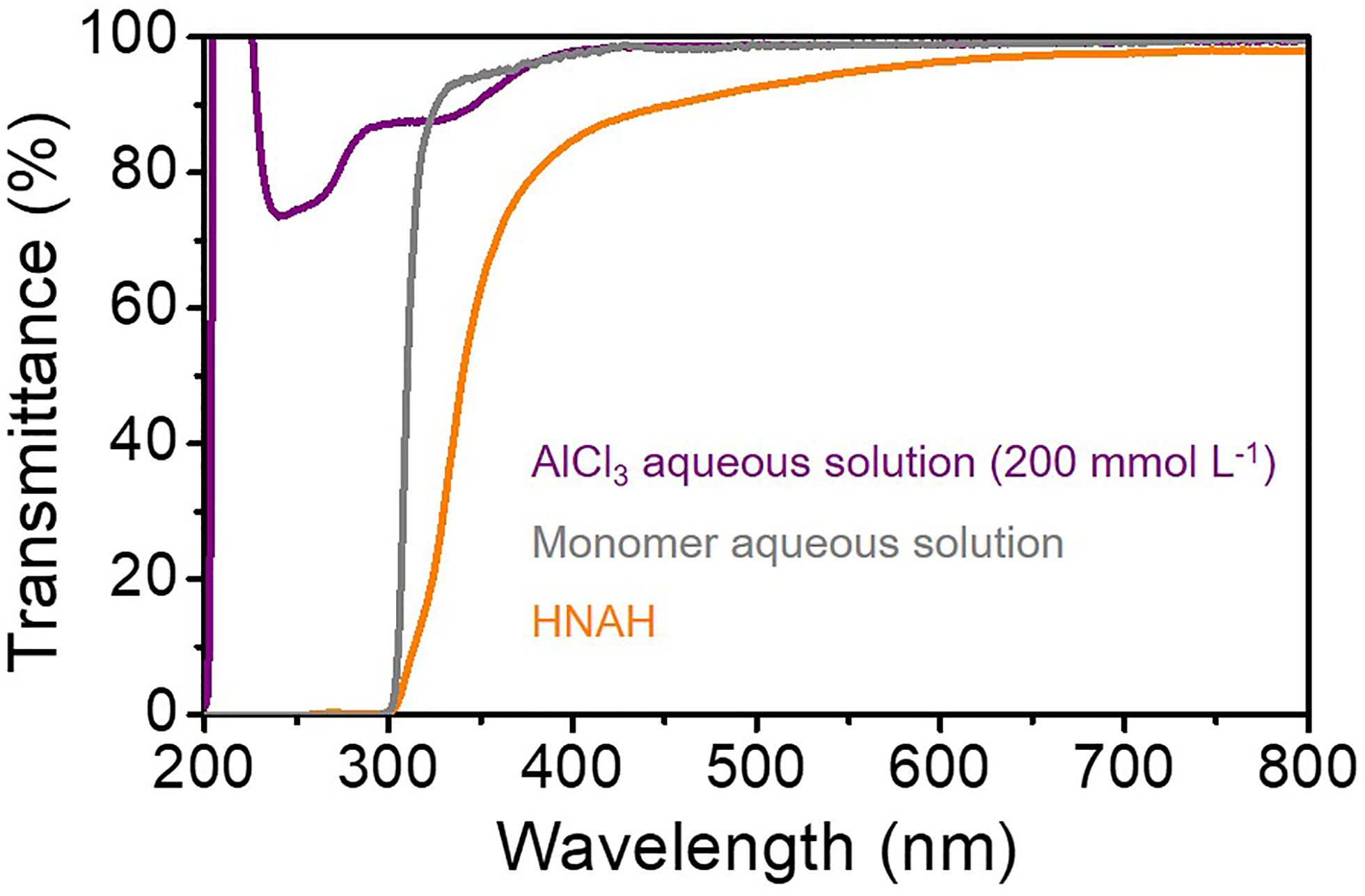


**Figure S11.** UV-Vis transmittance spectra of the AlCl_3_ aqueous solution, monomer aqueous solution and the HNAH.

**
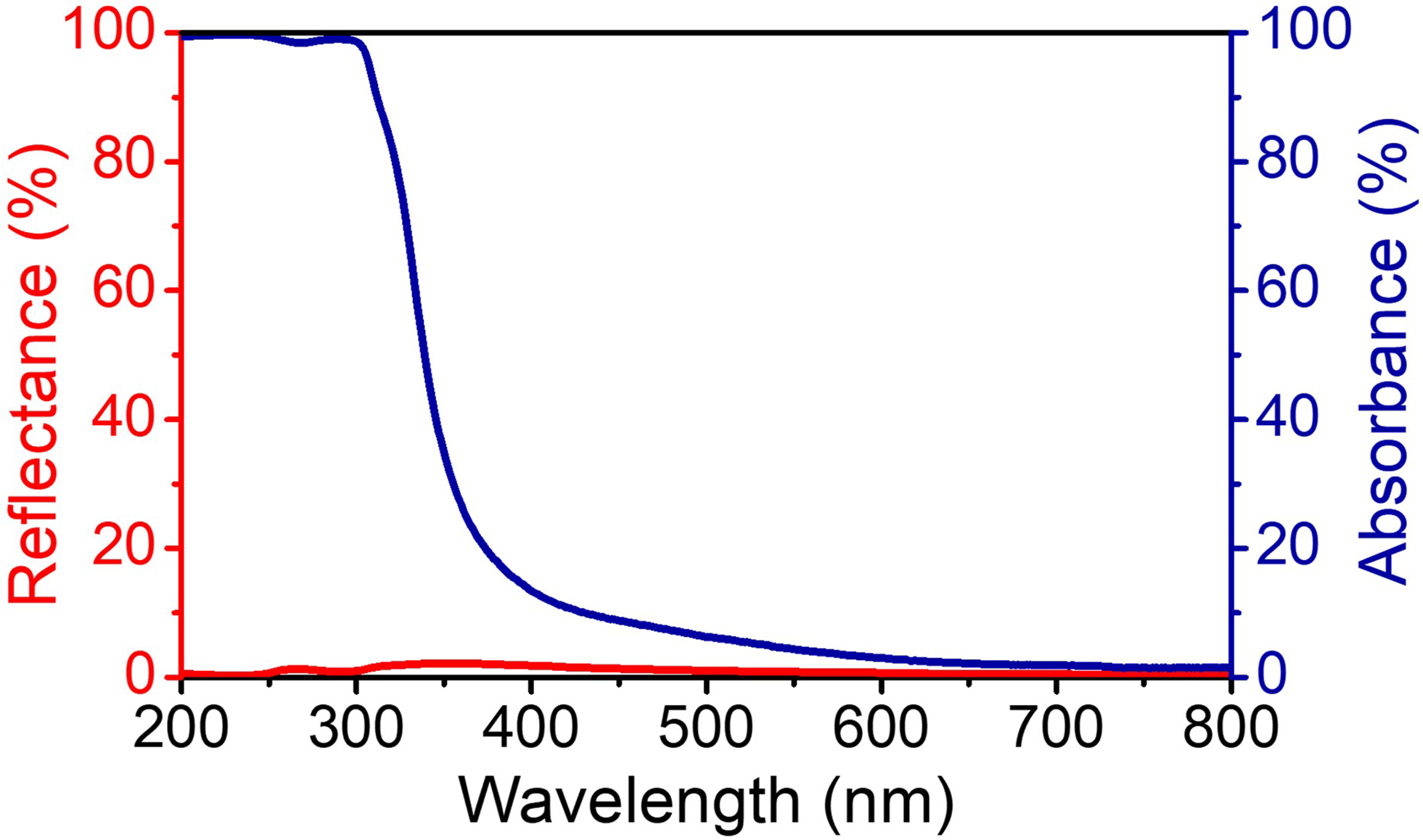
**

**Figure S12.** UV-Vis reflectance and absorbance spectra of HNAH.


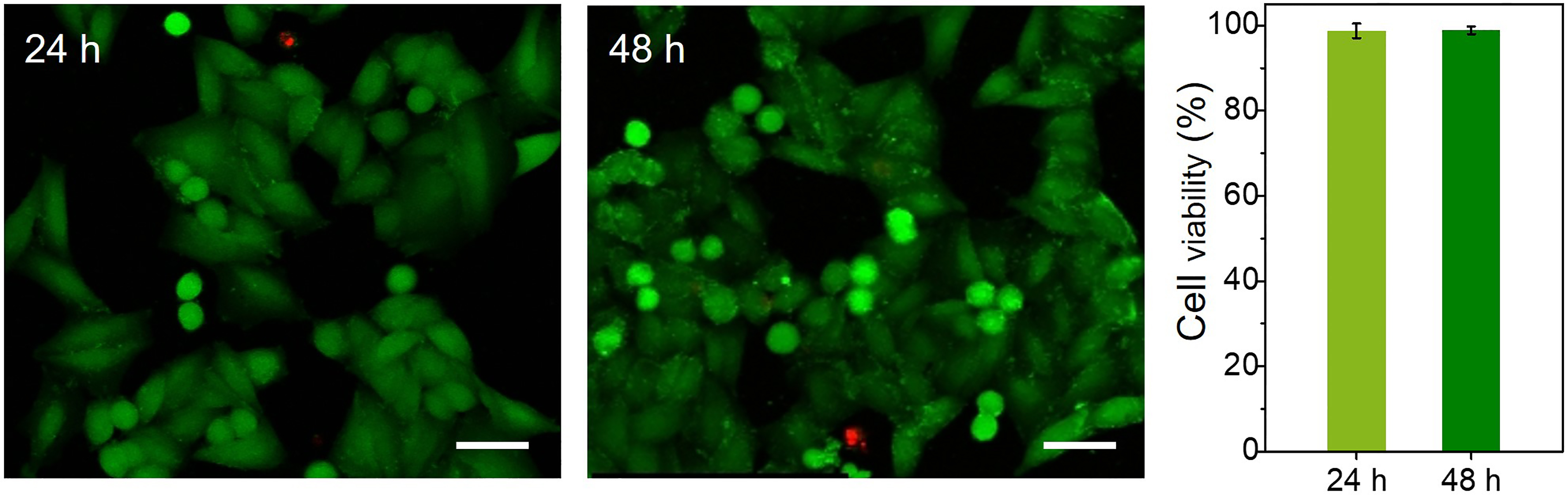


**Figure S13.** The confocal optical imaging and the cell viability of the cells in the control group with further cultured for 24 and 48 h.

**References**

1. K. J. Henderson, K. R. Shull, Effects of solvent composition on the assembly and relaxation of triblock copolymer-based polyelectrolyte gels. Macromolecules 45, 1631-1635 (2012).

2. I. Noda, Generalized two-dimensional correlation method applicable to infrared, raman, and other types of spectroscopy. Appl. Spectrosc. 47, 1329-1336 (1993).

3. I. Noda, Recent advancement in the field of two-dimensional correlation spectroscopy. J. Mol. Struct. 883, 2-26 (2008).

4. D. E. Rosenfeld, Z. Gengeliczki, B. J. Smith, T. D. P. Stack, M. D. Fayer, Structural dynamics of a catalytic monolayer probed by ultrafast 2D IR vibrational echoes. Science 334, 634-639 (2011).

5. I. Noda, 2DCOS and I three decades of two-dimensional correlation spectroscopy. J. Mol. Struct. 1124, 3-7 (2016).

6. S.-t. Sun, P.-y. Wu, Spectral insights into microdynamics of thermoresponsive polymers from the perspective of two-dimensional correlation spectroscopy. Chin. J. Polym. Sci. 35, 700-712 (2017).

7. I. Noda, Close-up view on the inner workings of two-dimensional correlation spectroscopy. Vib. Spectrosc. 60, 146-153 (2012).

8. I. Noda, Determination of two-dimensional correlation spectra using the Hilbert transform. Appl. Spectrosc. 54, 994-999 (2000).

9. G. Beaucage, Approximations leading to a unified exponential power-law approach to small-angle scattering. J. Appl. Cryst. 28, 717-728 (1995).

10. B. H. Hammouda, D. L.; Kline, S., Insight into clustering in poly(ethylene oxide) solutions. Macromolecules 37, 6932-6937 (2004).
